# Multisensory Input Modulates P200 and L2 Sentence Comprehension: A One-Week Consolidation Phase

**DOI:** 10.3389/fpsyg.2021.746813

**Published:** 2021-09-20

**Authors:** Nasim Boustani, Reza Pishghadam, Shaghayegh Shayesteh

**Affiliations:** Department of English, Ferdowsi University of Mashhad, Mashhad, Iran

**Keywords:** sentence comprehension, emotioncy, memory consolidation, attention, P200, N400

## Abstract

Multisensory input is an aid to language comprehension; however, it remains to be seen to what extent various combinations of senses may affect the P200 component and attention-related cognitive processing associated with L2 sentence comprehension along with the N400 as a later component. To this aim, we provided some multisensory input (enriched with data from three (i.e., exvolvement) and five senses (i.e., involvement)) for a list of unfamiliar words to 18 subjects. Subsequently, the words were embedded in an acceptability judgment task with 360 pragmatically correct and incorrect sentences. The task, along with the ERP recording, was conducted after a 1-week consolidation period to track any possible behavioral and electrophysiological distinctions in the retrieval of information with various sense combinations. According to the behavioral results, we found that the combination of five senses leads to more accurate and quicker responses. Based on the electrophysiological results, the combination of five senses induced a larger P200 amplitude compared to the three-sense combination. The implication is that as the sensory weight of the input increases, vocabulary retrieval is facilitated and more attention is directed to the overall comprehension of L2 sentences which leads to more accurate and quicker responses. This finding was not, however, reflected in the neural activity of the N400 component.

## Introduction

The growing literature offers that sentence comprehension depends on the long-term memory retrieval operations and unification operations (Bastiaansen and Hagoort, 2006; Hagoort, 2013). The neurobiological underpinnings of the sentence comprehension process have been investigated through the well-suited event-related potential (ERP) tool. In this regard, the interpretations of elicited ERP components offer feedbacks on minute alterations of neural responses to stimuli and allow analyzers to delve into variations in neural mechanisms. For instance, N400, as the most prominent language-related ERP component, is sensitive to prediction and expectation functions (Dierks et al., 1994) as well as semantic processing of words and sentences (Kaan, 2007). Like N400, P200 is sensitive to diverse language-oriented stimuli, yet its functions are more directed toward attention. To examine the functions of the P200 component, researchers (e.g., Luck and Hillyard, 1994; Federmeier and Kutas, 2002; Liu et al., 2003; Federmeier et al., 2005; Guo et al., 2012) have used various tasks. A general assumption is that the P200 component indexes higher-order perceptual processing modulated by language and attention (Luck and Hillyard, 1994; Lijffijt et al., 2009). Neuroscientists have evidenced that P200 characteristics are biased by attentional mechanisms in the influence of several factors, such as sentential context (Evans and Federmeier, 2007), emotion (Carretié et al., 2001), repetition (Schweinberger and Neumann, 2016), and gender (Ito and Urland, 2003). Considering the influential factors in P200 modulation in association with attention mechanism, we seek to place emphasis on senses as a factor pertinent to higher-order cognitive functions (Dudai, 2004; Matusz and Eimer, 2011) and specifically examine any possible interplay between senses and attention mechanism in integration with sentence comprehension. In this respect, investigations (e.g., Talsma and Woldorff, 2005; Talsma et al., 2010; Battich et al., 2020) have deciphered certain influences of senses on attention mechanism. In fact, coordination of multisensory input can take place across different stages of stimulus processing that are associated with, and can be changed by, attention (Talsma et al., 2010). On the one hand, stimulus-driven attention, which is drawn to the characteristics of inputs, can enhance memory via cognitive control mechanism (Wills et al., 2017); on the other hand, attention can facilitate the combination of multisensory input (Talsma et al., 2010). Hence, there are multifarious interactions between multisensory integration and attention mechanism modifying “the firing rate of perceptual neurons” (Macaluso et al., 2016).

Senses, not only in isolation but also in combination, may potentially affect attention and effective comprehension. It is basically assumed that different combinations of senses might capture different degrees of attention, as a result of which the accuracy and speed of comprehension may change. Several lines of research (e.g., Sparks et al., 1998; Stein et al., 2010; Aral and Sağlam, 2016; De Niear et al., 2016; Schneider and Kulmhofer, 2016; Myréen, 2017; Broadbent et al., 2018; Holler and Levinson, 2019; Jajarmi and Pishghadam, 2019) converge to suggest that the quality of input is associated with the characteristics of sensory representations, which are likely to take pivotal functions in how sensory signals cooperate with each other (Azamnouri et al., 2020; Shayesteh et al., 2020). Although there have been recent ERP studies addressing the role of sense combinations in overall L2 sentence comprehension (e.g., Shayesteh et al., 2020; Pishghadam et al., 2021b), there is only one single study that has investigated the role of multiple senses in the attention-related P200 component (Shayesteh, 2019). Based on the results of the study, there exists no change in the P200 amplitude if the input contains information from the combination of three or five senses. However, considering the importance of sensory combinations in the quality of inputs, the effect may manifest itself when some consolidation takes place. Given that Shayesteh (2019) verified the P200 potential in response to the main effect of the combination of senses with a short time interval (1 h) between receiving the input and performing on the comprehension task, we set out to do the experiment with 1-week interval (McDaniel et al., 1987; Dudukovic and Knowlton, 2006; Sobel et al., 2011; Baumann et al., 2019) hypothesizing that the consolidation of input might bolster variations in the effect of different sense combinations. The logic behind selecting this time interval was based on the neuroanatomical models of memory for the system consolidation process (Dudai, 2004). During this process, multiple neural networks coactivate, and memory goes through a reorganization process (McClelland et al., 1995). Due to its time-taking nature, it is believed that the process benefits the long-term retention of information 1 week after receiving the input (Wirebring et al., 2015; Ezzyat et al., 2018).

To address the core assumption of the current study and provide multisensory input, we chose a list of L2 vocabulary items about which the subjects had no previous knowledge. L2 was selected since all the similar studies were conducted in the subjects’ foreign language (Shayesteh et al., 2020; Pishghadam et al., 2021a, b). It is also believed that the emotional responses which can influence any kind of cognitive processing are absent in individuals’ non-native languages (Dylman and Bjärtå, 2018). Following Pishghadam et al. (2021b) and Shayesteh et al. (2020), we elaborated on the meaning and features of the selected items in a session we called the instruction session. To control the weight of senses in giving the instructions, we relied on previous behavioral (e.g., Pishghadam and Abbasnejad, 2016; Pishghadam and Shayesteh, 2016; Pishghadam et al., 2018) and electrophysiological (Shayesteh et al., 2020; Pishghadam et al., 2021a, b) studies and used Pishghadam (2016) sensory-based model of emotioncy (emotion + frequency) which exclusively illustrates different combinations of senses. The model sets the ground for discriminating a blend of visual, auditory, and kinesthetic/tactile senses (coined as exvolvement) from a combination of auditory, visual, kinesthetic/tactile, olfactory, and gustatory modalities (coined as involvement). Preceded by these levels, there is another level (coined as avolvement) in which the individuals have no sensory information about different concepts. When individuals transcend from avolvement to exvolvement, they use a limited number of sensory modalities. The nature of these modalities creates indirect sensory experiences of a concept which help them paint a blurry picture of reality. When the move is from exvolvement to involvement, more senses are involved and individuals’ conceptions of the world become more vivid and much closer to reality. That is why sensory experiences are believed to shape one’s cognition by degrees (Pishghadam, 2016). Based on these assumptions, the sensory-based model of emotioncy could contribute to the development of this study. Therefore, the model was used to teach a list of L2 vocabulary items to the subjects. To measure comprehension, the instructed vocabulary items were embedded in a sentence acceptability judgment task (designed based on Hagoort et al., 2004; Hald et al., 2006; Kos et al., 2012; Shayesteh et al., 2020; Pishghadam et al., 2021b), constituting pragmatically correct and incorrect sentences. While the subjects were doing the task, their electrical brain activity was being recorded.

Taken together, we presume that, in line with the previous studies, different combinations of senses affect attention-related cognitive processing as well as later stages of comprehension mechanism, and thus influence the behavioral responses (response time and response accuracy) of the individuals to the acceptability judgment task as well. To be specific, since retention is facilitated when the input is rich with sensory stimuli, more attention is paid to judge the acceptability of the sentences, resulting in more accurate responses. Increased attention improves the speed of comprehension and reacting to the acceptability of the sentences at the same time. Moreover, based on the prior literature, we assume that P200, as a major indicator of attention during sentence comprehension, may be modulated by the combination of senses subsequent to a 1-week memory consolidation phase. In particular, improving the sensory quality of input from avolvement to exvolvement and involvement, may show some meaningful changes in the P200 amplitude. To elaborate, enriched sensory input as a result of combining five senses leads to better and faster retention of the instructed word; thus more attention is directed to the meaning of the whole sentence (rather than the instructed word) for accurate comprehension. Therefore, involvement is expected to elicit a more positive effect in comparison with exvolvement and avolvement.

Moreover, to investigate if the probable sense combination effects continue to affect the later stages of sentence comprehension as well, we examine the N400 component as an index of semantic processing. According to Shayesteh (2019) and Shayesteh et al. (2020), the combinations of three and five senses do not differently modulate the negativity of the N400 in response to the comprehension of pragmatically correct and incorrect sentences. Yet, we hypothesize that the 1-week consolidation period between the instruction and the ERP recording may exert some changes to the previous findings. This may manifest itself in generating the smallest N400 amplitude in response to the sentences with involved target words. The input from the combination of five senses diminishes the semantic access difficulty, hence reducing the N400 effect.

## Methodology

### Subjects

Among the 23 volunteers who took part in the experiment, 3 were excluded since they did not meet the inclusion criteria (see section “Inclusion Criteria”). The electroencephalography (EEG) signals of 20 subjects (7 males and 13 females) were analyzed. Of the 20 subjects, two were excluded from the final analysis due to excessive muscle and eye movement artifacts. The subjects had no neurological impairment and were with normal or corrected-to-normal vision. They were right-handed adults (Oldfield, 1971), aged between 18 and 30, with normal or corrected-to-normal vision. They were native Persian speakers with intermediate English language levels (Allan, 1992) and normal working memory (Corsi, 1972).

### Inclusion Criteria

#### Pre-tests

To ensure homogeneity, several criteria were administered. Considering English language proficiency level, we used Oxford Quick Placement Test (Allan, 1992) to find subjects with intermediate English language level (the score of 30–40 out of 60). To check the subjects’ hand laterality, we accepted those volunteers whose Edinburgh Inventory of Handedness score (Oldfield, 1971) was within the 10–12 range. To assess their working memory, we conducted the computerized version of Corsi block-tapping test (Corsi, 1972). Drawing on Kessels et al. (2000) finding, we considered 5–6 as the average of Corsi Span for normal working memory. To practically measure the subjects’ familiarity with the words, Borsipour (2016) emotioncy scale was adapted. Based on the scores, we excluded those volunteers who had prior familiarity with any of the target words.

### Target Words

**FIGURE 1:**
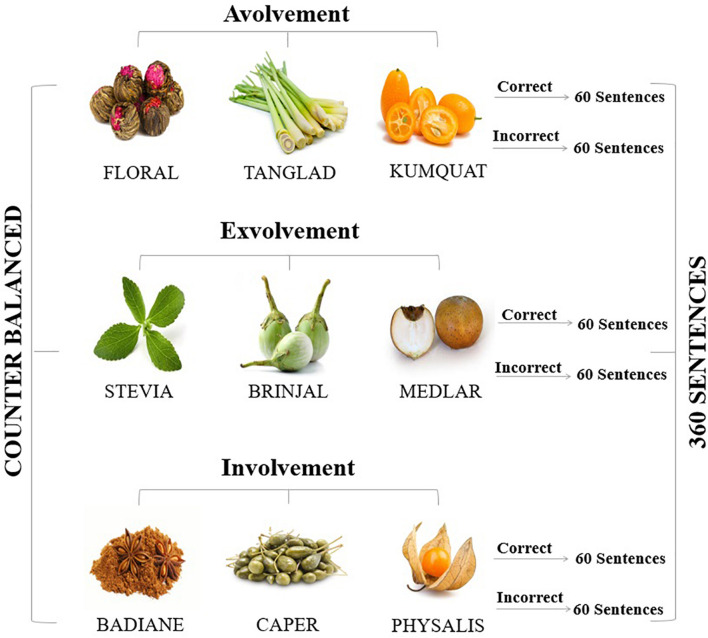
Sample instruction for various sense combinations.

To select the target words, we initially applied a article and pencil method and listed 30 English words (including plants, fruits, and vegetables) along with their Persian translation. We distributed the list of words among 130 respondents (73 female and 57 male), who were not the subjects of the study, and asked them to rate their familiarity with the words (Borsipour, 2016). From the 30-item list, 93% of the respondents had no familiarity with some of the words: Floral, stevia, tanglad, badiane, physalis, kumquat, medlar, brinjal, and caper. Consequently, these 9 words were selected as the target words for multisensory input and instruction (see Figure 1).

### Stimulus Materials

Drawing upon previous studies (Hagoort et al., 2004; Hald et al., 2006; Kos et al., 2012), we arranged a sentence acceptability judgment task consisting of 360 sentences (180 correct and 180 pragmatically incorrect, see Table 1) with a tripartite division of the target words (each containing 5 to 8 characters and 2 to 3 syllables) into avolvement, exvolvement, and involvement levels. We constructed 40 sentences (20 correct and 20 pragmatically incorrect) for each target word. The target words were all embedded in the sentence initial position. Moreover, to enhance the reliability of measures, the length (3–7 words) and complexity (simple present tense, singular type, and active voice) of all sentences were taken into account. We asked the subjects to judge the acceptability of the sentences according to the instruction they received in the instruction session.

**TABLE 1:** Examples of the correct and pragmatically incorrect sentences.

Sentence type	Condition	Example
Target	Correct	**Badiane** is brown
	Incorrect	**Badiane** is red

*The target words are boldfaced.*

### Procedure

The whole experiment consisted of two phases: the instruction phase and the experiment phase, which were a week apart. That is, each subject received the multisensory input during the instruction phase and came back to the lab for ERP recording a week after the instruction.

#### The Instruction

**FIGURE 2:**
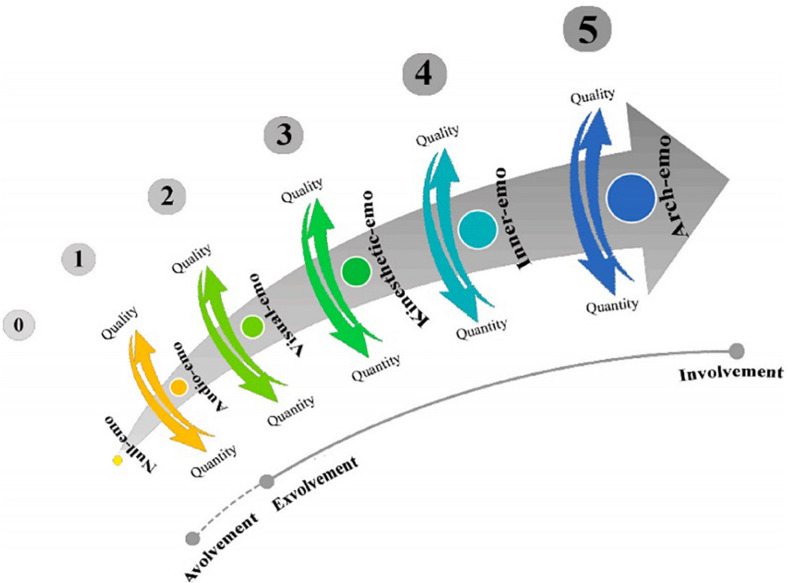
Emotioncy levels (Reprinted with permission from “Emotioncy, extraversion, and anxiety in willingness to communicate in English,” by Pishghadam, 2016, Proceedings of the 5th International Conference on Language, Education, and Innovation. London, United Kingdom).

Relying upon Pishghadam (2016) emotioncy model (Figure 2) and in accordance with Shayesteh et al. (2020) and Pishghadam et al. (2021b) studies, we came up with different forms of instruction to control the multisensory input the subjects were exposed to.

In the instruction session, each participant received a 30-min instruction for 6 out of 9 target words. Three words did not have any instruction since they were placed in the avolvement group. It is noteworthy that, for the instruction, we used real vegetables, plants, and fruits as well as a photo booklet and a PowerPoint to present the target words. The words were organized into 3 groups (G1, G2, and G3), and each participant received various sensory instructions depending on the group to which they belonged. To delineate the instruction in a more clarified manner, an example is provided (Table 2).

**TABLE 2:** Emotioncy instruction.

Group	Avolvement	Exvolvement	Involvement
G1	Floral	Stevia	Badiane
	Tanglad	Brinjal	Caper
	Kumquat	Medlar	Physalis

As Table 2 presents, the subjects did not receive any instruction for floral, tanglad, and kumquat (avolvement). At the exvolvement level, they received auditory, visual, and tactile information about stevia, brinjal, and medlar. Then, at the involvement level, they received full sensory involvement (auditory + visual + tactile + olfactory + gustatory) for badiane, caper, and physalis (see Table 3 for a sample instruction).

**TABLE 3:** A Sample Instruction.

Word	Sense combinations	Instruction
1. Medlar	Exvolvement (Auditory, visual, and Kinesthetic)	Ok now … have a look at this fruit. This is called medlar. As you can see, it has brown skin … can you eat the skin? Aha right! The skin is edible. Now touch it. How does it feel? Absolutely right! The skin is soft. Ok now let’s go a bit further … take that knife and cut the fruit. Cool! Look at that … you see the flesh? The flesh is edible. Now, do you see the seeds? Find the seeds inside. You see there are a lot of seeds inside. Touch them … Are they soft or hard? Aha right. They are hard. Medlar smells fruity … And it actually tastes sour
2. Physalis	Involvement (Auditory, visual, kinesthetic, Smell, and taste) + Research	Now, look at this one. Let me introduce it to you. This fruit is called physalis. Aha … yes right physalis. You see … it is round and small. The whole fruit looks like a cherry tomato, doesn’t it? Look at the skin … It is orange. Can you eat the skin or do you have to peel it? Aha right … the skin is edible. There is also a papery cover around the fruit. Is this cover edible? No, it is not …. Now touch the skin. It feels soft. Come on let’s cut the fruit and see what is inside. Oh good … Do you see all these seeds? Let’s smell it … You see, physalis smells fruity. Do you like to taste it? Yeah … let’s go for it. How does it taste? Right, it tastes a bit sour. Ok now … you have 1 min to search this word online

#### The Event-Related Potential Task

**FIGURE 3:**
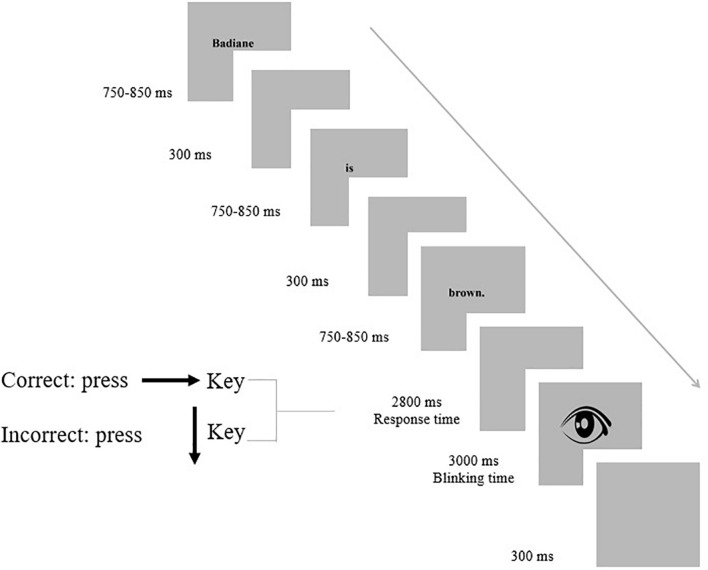
A sample ERP trial (Badiane is brown) in an experimental block of the task.

Following the procedure employed by similar studies (e.g., Kutas and Hillyard, 1980; Kos et al., 2012; Pishghadam et al., 2021b), the presentation method was in a word-by-word format (Figure 3). The font color was black, and the font size was Times New Roman 58 pt bold and sharp in the center of a 17-inch computer screen. Text covered a visual angle of 3° horizontally and 0.5° vertically at the center of a cool gray background. The sentences were randomized into 5 experimental blocks. Each block consisted of 72 trials, each taking 10 min, and then followed by a 5 min break. To design the task, we used Psychophysics Toolbox Version 3 (PTB-3) for MATLAB (version 2015a, The MathWorks, MA, United States). The subjects were required to judge the acceptability of each sentence by pressing the defined keyboard keys during response time.

### EEG Recording

The experiment phase was organized after a 1-week interval in an EEG lab. The subjects sat in a comfortable chair positioned ∼100 cm from a computer screen and were tested in a sound-attenuated and dimly lit chamber. Before the EEG recording, the subjects were instructed about the test and resting times (pauses between the blocks). Prior to commencing the task, a block with 20 sample sentences was presented to the subjects to get acquainted with the task. The lab was equipped with a 32-channel wireless g.Nautilus EEG system (gtec, Austria). The electrodes (Fz, FCz, Cz, Pz, Oz, AF3/4, F3/4/7/8, FT7/8, FC3/4, C3/4, P3/4/7/8, and PO7/8) were positioned on the cap based on the international 10–20 montage and in agreement with previous experiments (e.g., Hagoort et al., 2004; Van Berkum et al., 2005; Hald et al., 2006). Electrode impedances for all electrodes were kept below 5 kΩ. EEG signals were filtered by a notch filter of 50 Hz to eliminate AC line noise.

### Data Analysis

The recorded data were imported into MATLAB software toolbox (version 2015a, The MathWorks, MA, United States). To analyze waveforms, we used EEGLAB 13_6_5b (an extension of MATLAB software). EEG data were digitally filtered between 0.5–60 Hz and were re-referenced offline to the algebraic average of linked-mastoids. The Artifact Subspace Reconstruction (ASR) algorithm was applied to filter out noises; the remaining high frequencies were low-pass-filtered at 25 Hz. Epochs were calculated from 200 ms before to 1100 ms after the stimulus (i.e., the final word of the sentences) onset to check comprehension. Data deviations below −70 μVs and above + 70 μVs were excluded from the analysis. Drifts were removed according to a linear detrend algorithm (employing the 200 ms before the stimulus onset to 3 s after the stimulus). The mean was analyzed in a latency window of 180–280 ms and 300–550 ms for the P200 and the N400 components, respectively.

Then, IBM SPSS Statistics software (version 25) was run to examine the amplitudes and significance of contrasts resulted from the effects of the variables. The normality of mean amplitudes was examined by the classical Kolmogorov-Smirnov test, and an alpha level of 0.05 was applied in the *post hoc* analysis with Bonferroni correction. To explicate the Within-Subjects Effect tests and to protect against the probability of making a Type I error, *p* values were adjusted and the Greenhouse-Geisser correction was reported.

## Results

### Behavioral Data

To examine the subjects’ response accuracy (RA) and response time (RT), their behavioral performance across different combinations of senses (i.e., avolvement, exvolvement, and involvement), and linguistic condition (i.e., pragmatically correct and incorrect sentences) were assessed. Table 4 describes means and standard deviations.

**TABLE 4:** Mean values (M) and the standard deviations (SD) for RA and RT.

Sense combination	Response accuracy				Response time (s)			
	Correct		Incorrect		Correct		Incorrect	
	*M*	*SD*	*M*	*SD*	*M*	*SD*	*M*	*SD*
Avolvement	5.89 (9%)	9.99	6.39 (10%)	10.01	2.73	0.37	2.69	0.41
Exvolvement	38.94 (64%)	11.38	39.72 (66%)	11.95	0.99	0.23	1.09	0.24
Involvement	46.56 (77%)	10.43	44.33 (73%)	9.24	0.93	0.19	1.00	0.21

#### Response Accuracy

A two-way repeated-measures ANOVA was conducted to examine the subjects’ performances in judging the acceptability of pragmatically correct and incorrect sentences based on different combinations of senses.

The results showed the significant main effect of sense combinations [*F*(2,34) = 77.29, *p* < 0.05, η^2^ = 0.82, 1-β = 1.00]. The Pairwise *post hoc* results revealed that the subjects outperformed the sentences with involved words (*M* = 45.44) in comparison to the sentences with exvolved (*M* = 39.33) and avolved (*M* = 6.139) words. Specifically, there was a significant difference between avolvement and exvolvement (*MD* = −33.19, *p* < 0.001), between avolvement and involvement (*MD* = −39.30, *p* < 0.001), and between exvolvement and involvement (*MD* = −6.11, *p* < 0.01). As a result, the subjects’ comprehension and judgment did meaningfully vary across different combinations of senses (i.e., avolvement < exvolvement < involvement).

While the main effect of sense combinations was significant, its interaction with linguistic condition was not significant [*F*(2,34) = 1.34, *p* > 0.05, η^2^ = 0.17, 1-β = 0.77].

#### Response Time

Given that there were 22 missing RT values for correct and pragmatically incorrect sentences with avolved target words, we excluded avolvement and established a two-way repeated measures ANOVA.

The F-test results presented significant differences for different combinations of senses [*F*(1,17) = 9.06, *p* < 0.05, η^2^ = 0.34, 1-β = 0.81]. The pairwise *post hoc* results showed a significant difference between exvolvement and involvement (*M* = 0.07, *p* < 0.01). More specifically, the subjects’ reaction times to the sentences with involved words (*M* = 0.97 s) were faster than those with exvolved words (*M* = 1.04 s). Data conveys that different combinations of senses had a noteworthy impact on the subjects’ mental agility to judge the acceptability of the sentences.

### Electrophysiological Data

**FIGURE 4:**
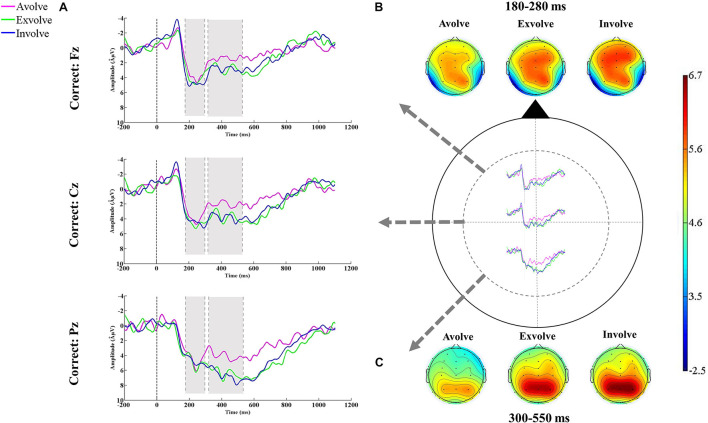
**(A)** Grand-average ERPs (*N* = 18) elicited by the correct condition in response to different combinations of senses (i.e., avolvement, exvolvement, and involvement. **(B)** The topographic maps plotted in the latency window of 180–280 ms after the stimulus onset. **(C)** The topographic maps plotted in the latency window of 300–550 ms after the stimulus onset.

**FIGURE 5:**
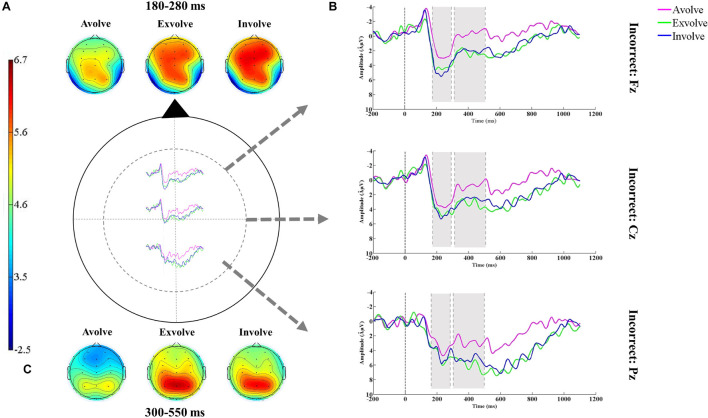
**(A)** The topographic maps plotted in the latency window of 180–280 ms after the stimulus onset. **(B)** Grand-average ERPs (*N* = 18) elicited by the pragmatically incorrect condition in response to different combinations of senses (i.e., avolvement, exvolvement, and involvement). **(C)** The topographic maps plotted in the latency window of 300–550 ms after the stimulus onset.

Sentence comprehension gave rise to a widely distributed positive-going deflection in the 180–280-ms time window, typical of the P200 component, across correct and pragmatically incorrect sentences. This component was followed by a later negativity in the time window of 300–550 ms which resembled the classic N400 effect (see Figures 4, 5). The mean amplitudes of the P200 and the N400 components were analyzed over the frontal (Fz), central (Cz), and parietal (Pz) sites. The reason we put these three electrodes into analysis was that the small number of electrodes is evidenced to decrease Type I error in the analysis (Tanner et al., 2017). Moreover, drawing upon previous studies (Tanner et al., 2014, 2017; Pishghadam et al., 2021a) the selected electrodes used in the analyses evince maximum efficacy to give a detailed account of the ERP components’ outcomes in sentence comprehension.

#### The 180–280-ms Time Window

A three-way repeated measures ANOVA (with sense combination (3), linguistic condition (2), and electrode (3) as the independent variables) was conducted to compare the mean amplitudes of the P200 waveform. Although tests showed a significant effect for sense combination [*F*(2,34) = 11.82, *p* < 0.05, η^2^ = 0.41, 1-β = 0.99], sense combination × channel [*F*(4,68) = 2.86, *p* > 0.05, η^2^ = 0.14, 1-β = 0.82] and sense combination × linguistic condition [*F*(2,34) = 2.79, *p* ≤ 0.05, η^2^ = 0.14, 1-β = 0.81] had no significant effect on P200 modulations.

The estimation of mean values presented variations across different combinations of senses: avolvement (*M* = 3.17 μV), exvolvement (*M* = 5.08 μV), and involvement (*M* = 6.58 μV). The F-test value displayed that sense combination was a significant factor in modulating the P200 potential. Furthermore, the *post hoc* comparisons differentiated between the P200 amplitude for avolvement and exvolvement (*p* < 0.01), avolvement and involvement (*p* < 0.01), and exvolvement and involvement (*p* < 0.05). In sum, the sentences with involved target words elicited the largest P200 amplitude.

In order to examine if there is any relationship between the magnitude of the P200 and the behavioral responses (i.e., RA and RT), we calculated Spearman’s correlation coefficient. Since, according to the three-way repeated measures ANOVA, electrode did not have a significant main effect on the amplitude of the P200, the average of the amplitudes for the three electrodes was correlated with the RA and RT estimates. As Table 5 shows, the correlation coefficients between P200 and RA and P200 and RT indexes varied from ρ = 0.20 to ρ = 0.37 and ρ = −15 to ρ = −17, respectively. To explicate, as the RA rate to the correct and pragmatically incorrect sentences increased under avolved, exvolved, and involved conditions, the P200 amplitude turned out to be more positive. However, with an increase in the RT rate, the P200 became smaller in size. Therefore, it can be concluded that the P200 amplitude increases when the subjects give more accurate responses to the sentences, straight away.

**TABLE 5:** The correlational analysis for the magnitude of the P200 and the RA and RT estimates.

		RA						RT				P200					
		AvC	AvP	ExC	ExP	InC	InP	ExC	ExP	InC	InP	AvC	AvP	ExC	ExP	InC	InP
RA	AvC	1															
	AvP	0.93^∗∗^	1														
	ExC	−0.40	−0.40	1													
	ExP	−0.57^∗^	−0.56^∗^	0.82^∗∗^	1												
	InC	−0.35	−0.45	0.68^∗∗^	0.50^∗^	1											
	InP	−0.66^∗∗^	−0.70^∗∗^	0.72^∗∗^	0.77^∗∗^	0.66^∗∗^	1										
RT	ExC	0.12	0.09	−0.57^∗^	−0.59^∗∗^	−0.27	−0.17	1									
	ExP	0.25	0.22	−0.48^∗^	−0.62^∗∗^	−0.14	−0.17	0.87^∗∗^	1								
	InC	0.26	0.34	−0.63^∗∗^	−0.43	−0.71^∗∗^	−0.34	0.71^∗∗^	0.52^∗^	1							
	InP	0.19	0.20	−0.70^∗∗^	−0.70^∗∗^	−0.40	−0.42^∗^	0.80^∗∗^	0.79^∗∗^	0.66^∗∗^	1						
P200	AvC	0.35^∗^	0.36	−0.50^∗^	−0.46	−0.51^∗^	−0.39	0.31	0.27	0.46	0.50^∗^	1					
	AvP	0.34	0.37^∗∗^	−0.59^∗∗^	−0.38	−0.36	−0.30	0.24	0.13	0.41	0.38	0.38	1				
	ExC	−0.18	−0.21	0.26^∗^	−0.32	−0.04	−0.24	−0.16^∗∗^	−0.02	−0.21	0.27	0.11	0.27	1			
	ExP	−0.04	−0.09	−0.20	0.20^∗^	−0.37	−0.20	0.09	−0.15^∗^	0.15	0.29	0.24	0.09	0.52^∗^	1		
	InC	−0.10	−0.10	0.11	−0.02	0.21^∗^	−0.03	−0.14	−0.23	−0.17^∗^	−0.06	0.00	−0.04	0.57^∗^	0.68^∗∗^	1	
	InP	0.04	0.00	−0.20	−0.30	−0.18	0.20^∗∗^	−0.02	0.01	−0.14	−0.15^∗^	0.23	0.27	0.66^∗∗^	0.78^∗∗^	0.61^∗∗^	1

**Correlation is significant at the 0.01 level (two-tailed).

*Correlation is significant at the 0.05 level (two-tailed).

*AvC, avolve-correct; AvP, avolve-pragmatic; ExC, exvolve-correct; ExP, exvolve- pragmatic; InC, involve-correct; InP, involve- pragmatic.*

#### The 300–550-ms Time Window

To delineate the effects of the variables on the N400 potential, a three-way repeated measures ANOVA (with sense combination (3), linguistic condition (2), and electrode (3) as the independent variables) was conducted. Tests revealed a significant effect for sense combination [*F*(2,34) = 16.41, *p* < 0.05, η^2^ = 0.49, 1-β = 0.99].

Considering the mean values for the avolved (*M* = 1.85 μV), exvolved (*M* = 5.01 μV), and involved (*M* = 6.19 μV) types, the *F*-test value showed that sense combination was a significant factor in modulating the N400 potential. The *post hoc* comparisons differentiated between the N400 amplitude for avolvement and exvolvement (*MD* = −3.15 μV, *p* < 0.01), avolvement and involvement (*MD* = −4.33 μV, *p* < 0.01), but did not distinguish between exvolvement and involvement (*MD* = −1.17 μV, *p* > 0.01). In sum, there were no significant differences between the sentences with exvolved and involved target words in modifying the N400 potential.

## Discussion

To discover the behavioral and cortical responses to various combinations of senses, we used Pishghadam (2016) sensory-based model of emotioncy and checked L2 sentence comprehension. In what follows, we delineate the behavioral and electrophysiological results elicited by different combinations of senses as a result of a 1-week consolidation phase.

### Behavioral Findings

The obtained results confirmed our first hypothesis acknowledging that the meaningful effect of the combinations of senses (i.e., avolvement, exvolvement, and involvement) on the comprehension mechanism improved RA and reduced RT. That is, the significant differences in the number of correct judgments and the time they spent on each item, across the combination of no sense, three senses, and five senses, provided pieces of strong evidence that the tripartite architecture of the instruction was significantly effective. Several experiments have similarly shown the positive impact of multiple senses on RA and RT (e.g., Frassinetti et al., 2002; Lovelace et al., 2003; Bolognini et al., 2005; Shayesteh, 2019; Shayesteh et al., 2020). In particular, the enriched sensory input from the combination of five senses enhances attention-related cognitive processing and comprehension mechanism, empowering the subjects’ performance on the acceptability judgment task. The results are specifically consistent with the findings of Shayesteh (2019) and Shayesteh et al. (2020), which evidenced the significant influence of sense combinations on RA. However, in contrast to their reports, the interaction effect of sense combination and linguistic condition was not significant. That is, in their study, different combinations of senses affected the comprehension of pragmatically incorrect items and not the correct ones, whereas in the current study, senses affected the comprehension of both correct and pragmatically incorrect items. One justification could be that, for the correct items, all instructed words (i.e., exvolved and involved ones) could be equally and accurately recalled after a 1-h interval (see Shayesteh, 2019); yet, after a week, due to the concurrent effect of forgetting and memory consolidation, recalling depended more on the sensory weight of the input. During this 1-week interval, some forgetting takes place, which is a natural process in knowledge acquisition. At the same time, the consolidation process, as a result of the neocortical integration of input into the mental lexicon, yields a “truly word-like behavior” (Bakker et al., 2015). Therefore, it is basically assumed that an extended memory consolidation period is required for the words to undergo the lexicalization process and be completely lexicalized. Such an account provides the implication that sensory enriched input is more resistant to forgetting and more probable to consolidate. Accordingly, to delay forgetting and maximize comprehension, the multisensory input needs to have integrated data from the combination of the five senses.

Moreover, in accordance with the literature (e.g., Shayesteh, 2019; Shayesteh et al., 2020; Pishghadam et al., 2021b,a), significant changes in the behavioral responses were also evident in RT as a result of different combinations of senses. The findings led us to characterize the combination of five senses (more than the combination of the three senses) as an encouraging factor in providing quick responses to the correct and pragmatically incorrect items. For the sentences with avolved target words, the subjects either had the longest hesitation to respond to an item or skipped the item due to a lack of sensory knowledge. Overall, retention is facilitated when input is rich with sensory stimuli, and thus more attention is paid to the comprehension of the whole sentence rather than recalling the target words, resulting in more accurate and quicker responses.

### Electrophysiological Findings

#### The 180–280-ms Time Window

The electrophysiological results of the study showed that the P200 response is larger in comprehending the sentences with involved target words and smaller in comprehending the sentences with avolved and exvolved target words. Changes in the P200 amplitude conform to the second hypothesis and display that this component is significantly modified by enriched sensory input from the combination of five senses during the retrieval of information from long-term memory. Considering P200 as a major indicator of attention during sentence comprehension (Luck and Hillyard, 1994; Lijffijt et al., 2009; Shayesteh, 2019) we deduced that as the sensory weight of the input decreases from involvement to exvolvement and avolvement, more attention and internal concentration are required to recall the target word and, thus, less attention is directed to the overall comprehension of the sentence due probably to higher cognitive load. To be specific, when the subjects were confronted with avolved and exvolved (vs. involved) target words, their minds were busy retrieving the required information regarding its meaning and, therefore, were not able to easily concentrate on the meaning of the whole sentence. As a result, their response time and accuracy were negatively affected. This conclusion was further reinforced by the correlation results of P200 and behavioral estimates which revealed that the subjects’ slower and less accurate responses to the sentences decreased the positivity of the P200.

In brief, our findings revealed that sense combination was a significant factor in modulating the P200 potential. However, this result was not reported in Shayesteh (2019) experiment on sentence comprehension after a 1-h interval time between the instruction session and the subjects’ performance on the acceptability judgment task. A probable justification could reside in the neural transformations which occur during a 1-week consolidation phase and the interplay between forgetting and consolidation processes reinforcing variations in the effect of different sense combinations. In comparison with the 1-h interval, during the 1-week interval, much more forgetting and consolidation take place. Essentially, in the acquisition of information system, forgetting is the natural and inevitable cost of storage. Alternatively, memory consolidation, as a memory management mechanism, strengthens the memory traces and connections and leads to the formation of memory for words and automatic processing (Subagdja et al., 2012; Bakker et al., 2015). These brain processes encompass modifications in cell signaling during the retrieval of the accumulated information across the consolidation period (Davis and Zhong, 2017).

Our findings suggest that, although forgetting is inherent to knowledge acquisition, improvements in the sensory quality of input from avolvement to exvolvement and involvement may help ameliorate the effect, increase cognitive control and the degree of attention to the sentence (Wills et al., 2017), and eventually improve efficient retrieval and accurate comprehension. Overall, it is concluded that the more senses are involved in learning a new concept, the more probable the new information is committed to long-term memory and less susceptible to forgetting. To put it differently, sensory involvement exerts influences on the interaction between memory formation for newly acquired information, consolidation, and forgetting processes, which finally determine the comprehension of sentences. Moreover, modifications in the P200 potentials, whose responsiveness to attention mechanism is remarkable, seem to manifest some susceptibility to the retrieval of stored information with diverse multisensory inputs during a 1-week consolidation phase. As such, for successful long-term memory retrieval and appropriate comprehension, providing an opportunity for employing the five-sense combination can be a solution to internalize new information.

#### The 300–550-ms Time Window

Examining the electrical activities of the brain under the avolved, exvolved, and involved conditions, we observed an N400 component right after the P200, which was more obvious in sentences with avolved target words. In particular, the results did not completely confirm our third hypothesis and showed that the N400 response, as an indicator of semantic access (Hagoort et al., 2004; Kutas and Federmeier, 2011), was larger in comprehending the sentences with avolved target words rather than the ones with exvolved and involved words. Given that avolved target words were unknown to the subjects, they experienced more cognitive difficulty in comprehending the meaning of the pertinent sentences, which led to a larger negativity in the 300–550-ms time window. In effect, enrichment in the sensory quality of the input from avolvement to exvolvement and avolvement to involvement significantly enhanced access to the semantic memory. However, the combinations of three and five senses did not make distinctive alterations in the N400 amplitude, which may probably result from the short instruction time. The pattern of N400 changes, though similar to the findings of Shayesteh et al. (2020), was different from that of the P200 reflecting that the influence of sensory qualities of the input, after 1-week consolidation phase, might be restricted to the early stages of L2 sentence comprehension, notwithstanding that further research needs to be conducted for extended generalizability.

Last but not least, in order to have a more detailed insight into the characteristics of P200 and N400, complementary studies could be conducted to correlate these ERP responses with those of the target words. Correlating the results of P200 with other indicators of memory can add extra weight to the findings of this study. Besides, given that, due to the task limitation and time limits, we could use nine target words for the purpose of this experiment, future investigations are recommended to implement more vocabulary items. Additionally, a larger sample size will provide more reliable findings. Moreover, notwithstanding the advantages of the EEG with a perceptive temporal account, it is suggested to combine it with the findings of fMRI for an improved spatial representation. Finally, yet importantly, replication of this experiment in subjects’ first language will expand the findings.

## Data Availability Statement

The raw data supporting the conclusions of this article will be made available by the authors, without undue reservation.

## Ethics Statement

The studies involving human participants were reviewed and approved by the Ferdowsi University of Mashhad Ethics Committee (Code 1321). The patients/participants provided their written informed consent to participate in this study.

## Author Contributions

RP and SS conceived and designed the experiments, and reviewed and edited the manuscript. SS and NB performed the experiments and wrote the article. SS analyzed the data. RP, NB, and SS contributed reagents, materials, and analysis tools. All authors contributed to the article and approved the submitted version.

## Conflict of Interest

The authors declare that the research was conducted in the absence of any commercial or financial relationships that could be construed as a potential conflict of interest.

## Publisher’s Note

All claims expressed in this article are solely those of the authors and do not necessarily represent those of their affiliated organizations, or those of the publisher, the editors and the reviewers. Any product that may be evaluated in this article, or claim that may be made by its manufacturer, is not guaranteed or endorsed by the publisher.

## References

[B1] Allan D. (1992). *The Oxford Quick Placement Test.* Oxford: Oxford University Press.

[B2] Aral N. Sağlam M. (2016). “Sensory development in infants,” in *Current Advances in Education*, eds Atasoy E. Jażdżewska I. Yaldır H. (Sofia.: St. Klment Ohrdsk Unversty Press), 264–277.

[B3] Azamnouri N. Pishghadam R. Naji Meidani E. (2020). The role of emotioncy in cognitive load and sentence comprehension of language learners. *Issues Lang. Teach.* 9 29–55. 10.22054/ilt.2020.51543.485

[B4] Bakker I. Takashima A. van Hell J. G. Janzen G. McQueen J. M. (2015). Tracking lexical consolidation with ERPs: lexical and semantic-priming effects on N400 and LPC responses to newly-learned words. *Neuropsychologia* 79 33–41. 10.1016/j.neuropsychologia.2015.10.020 26476370

[B5] Bastiaansen M. Hagoort P. (2006). Oscillatory neuronal dynamics during language comprehension. *Prog. Brain Res.* 159 179–196. 10.1016/S0079-6123(06)59012-0 17071231

[B6] Battich L. Fairhurst M. Deroy O. (2020). Coordinating attention requires coordinated senses. *Psychonomic Bull. Rev.* 27 1–13. 10.3758/s13423-020-01766-z 32666194 PMC7704499

[B7] Baumann O. Crawshaw E. McFadyen J. (2019). Survival of the fittest: increased stimulus competition during encoding results in fewer but more robust memory traces. *Front. Psychol.* 10:21. 10.3389/fpsyg.2019.00021 30740071 PMC6357916

[B8] Bolognini N. Frassinetti F. Serino A. Làdavas E. (2005). “Acoustical vision” of below threshold stimuli: interaction among spatially converging audiovisual inputs. *Exp. Brain Res.* 160 273–282. 10.1007/s00221-004-2005-z 15551091

[B9] Borsipour B. (2016). *Emotioncy and Willingness to Read: A Case of Iranian EFL Learners.* Mashhad: Ferdowsi University of Mashhad. Unpublished master’s thesis.

[B10] Broadbent H. J. Osborne T. Rea M. Peng A. Mareschal D. Kirkham N. Z. (2018). Incidental category learning and cognitive load in a multisensory environment across childhood. *Dev. Psychol.* 54 1020–1028. 10.1037/dev0000472 29309181 PMC5961402

[B11] Carretié L. Mercado F. Tapia M. Hinojosa J. A. (2001). Emotion, attention, and the ‘negativity bias’, studied through event-related potentials. *Int. J. Psychophysiol.* 41 75–85. 10.1016/S0167-8760(00)00195-1 11239699

[B12] Corsi P. M. (1972). *Human Memory and the Medial Temporal Region of the Brain.* Quebec, QC: McGill University. Doctoral Thesis.

[B13] Davis R. L. Zhong Y. (2017). The biology of forgetting—a perspective. *Neuron* 95 490–503. 10.1016/j.neuron.2017.05.039 28772119 PMC5657245

[B14] De Niear M. A. Koo B. Wallace M. T. (2016). Multisensory perceptual learning is dependent upon task difficulty. *Exp. Brain Res.* 234 3269–3277. 10.1007/s00221-016-4724-3 27401473 PMC5073017

[B15] Dierks T. Frölich L. Ihl R. Maurer K. (1994). Event-related potentials and psychopharmacology. *Pharmacopsychiatry* 27 72–74. 10.1055/s-2007-1014282 7913237

[B16] Dudai Y. (2004). The neurobiology of consolidations, or, how stable is the engram? *Annu. Rev. Psychol.* 55 51–86. 10.1146/annurev.psych.55.090902.142050 14744210

[B17] Dudukovic N. M. Knowlton B. J. (2006). Remember–know judgments and retrieval of contextual details. *Acta Psychol.* 122 160–173. 10.1016/j.actpsy.2005.11.002 16405897

[B18] Dylman A. S. Bjärtå A. (2018). When your heart is in your mouth: the effect of second language use on negative emotions. *Cogn. Emot.* 33 1284–1290. 10.1080/02699931.2018.1540403 30384794

[B19] Evans K. M. Federmeier K. D. (2007). The memory that’s right and the memory that’s left: event-related potentials reveal hemispheric asymmetries in the encoding and retention of verbal information. *Neuropsychologia* 45 1777–1790. 10.1016/j.neuropsychologia.2006.12.014 17291547 PMC2758159

[B20] Ezzyat Y. Inhoff M. C. Davachi L. (2018). Differentiation of human medial prefrontal cortex activity underlies long-term resistance to forgetting in memory. *J. Neurosci.* 38 10244–10254. 10.1523/JNEUROSCI.2290-17.2018 30012697 PMC6262147

[B21] Federmeier K. D. Kutas M. (2002). Picture the difference: electrophysiological investigations of picture processing in the two cerebral hemispheres. *Neuropsychologia* 40 730–747. 10.1016/S0028-3932(01)00193-2 11900725

[B22] Federmeier K. D. Mai H. Kutas M. (2005). Both sides get the point: hemispheric sensitivities to sentential constraint. *Memory Cogn.* 33 871–886. 10.3758/BF03193082 16383175

[B23] Frassinetti F. Bolognini N. Làdavas E. (2002). Enhancement of visual perception by crossmodal visuo-auditory interaction. *Exp. Brain Res.* 147 332–343. 10.1007/s00221-002-1262-y 12428141

[B24] Guo T. Misra M. Tam J. W. Kroll J. F. (2012). On the time course of accessing meaning in a second language: an electrophysiological and behavioral investigation of translation recognition. *J. Exp. Psychol. Learn. Memory Cogn.* 38 1165–1186. 10.1037/a0028076 22686844 PMC3925769

[B25] Hagoort P. (2013). MUC (memory, unification, control) and beyond. *Front. Psychol.* 4:416. 10.3389/fpsyg.2013.00416 23874313 PMC3709422

[B26] Hagoort P. Hald L. Bastiaansen M. Petersson K. M. (2004). Integration of word meaning and world knowledge in language comprehension. *Science* 304 438–441. 10.1126/science.1095455 15031438

[B27] Hald L. A. Bastiaansen M. C. Hagoort P. (2006). EEG theta and gamma responses to semantic violations in online sentence processing. *Brain Lang.* 96 90–105. 10.1016/j.bandl.2005.06.007 16083953

[B28] Holler J. Levinson S. C. (2019). Multimodal language processing in human communication. *Trends Cogn. Sci.* 23 639–652. 10.1016/j.tics.2019.05.006 31235320

[B29] Ito T. A. Urland G. R. (2003). Race and gender on the brain: electrocortical measures of attention to the race and gender of multiply categorizable individuals. *J. Pers. Soc. Psychol.* 85 616–626. 10.1037/0022-3514.85.4.616 14561116

[B30] Jajarmi H. Pishghadam R. (2019). Emotioncy-Based language instruction: a key to enhancing EFL learners’ vocabulary retention. *Appl. Res. English Lang.* 8 207–226. 10.22108/are.2019.114399.1388

[B31] Kaan E. (2007). Event-related potentials and language processing: a brief overview. *Lang. Linguistics Compass* 1 571–591. 10.1111/j.1749-818X.2007.00037.x

[B32] Kessels R. P. C. van Zandvoort M. J. E. Postman A. Kapelle L. J. de Hand E. H. F. (2000). The corsi block-tapping task: standardization and normative data. *Appl. Neuropsychol.* 7 252–258. 10.1207/S15324826AN0704_8 11296689

[B33] Kos M. Van den Brink D. Hagoort P. (2012). Individual variation in the late positive complex to semantic anomalies. *Front. Psychol.* 3:318. 10.3389/fpsyg.2012.00318 22973249 PMC3434872

[B34] Kutas M. Federmeier K. D. (2011). Thirty years and counting: finding meaning in the N400 component of the event-related brain potential (ERP). *Ann. Rev. Psychol*. 62, 621–647. 10.1146/annurev.psych.093008.131123 20809790 PMC4052444

[B35] Kutas M. Hillyard S. A. (1980). Reading between the lines: event-related brain potentials during natural sentence processing. *Brain Lang.* 11 354–373. 7470854 10.1016/0093-934x(80)90133-9

[B36] Lijffijt M. Lane S. D. Meier S. L. Boutros N. N. Burroughs S. Steinberg J. L. (2009). P50, N100, and P200 sensory gating: relationships with behavioral inhibition, attention, and working memory. *Psychophysiology* 46 1059–1068. 10.1111/j.1469-8986.2009.00845.x 19515106 PMC2821570

[B37] Liu Y. Perfetti C. A. Hart L. (2003). ERP evidence for the time course of graphic, phonological, and semantic information in chinese meaning and pronunciation decisions. *J. Exp. Psychol. Learn. Memory Cogn.* 29 1231–1247. 10.1037/0278-7393.29.6.1231 14622057

[B38] Lovelace C. T. Stein B. E. Wallace M. T. (2003). An irrelevant light enhances auditory detection in humans: a psychophysical analysis of multisensory integration in stimulus detection. *Cogn. Brain Res.* 17 447–453. 10.1016/S0926-6410(03)00160-5 12880914

[B39] Luck S. J. Hillyard S. A. (1994). Electrophysiological correlates of feature analysis during visual search. *Psychophysiology* 31 291–308. 10.1111/j.1469-8986.1994.tb02218.x 8008793

[B40] Macaluso E. Noppeney U. Talsma D. Vercillo T. Hartcher-O’Brien J. Adam R. (2016). The curious incident of attention in multisensory integration: bottom-up vs. top-down. *Multisensory Res.* 29 557–583. 10.1163/22134808-00002528

[B41] Matusz P. J. Eimer M. (2011). Multisensory enhancement of attentional capture in visual search. *Psychonomic Bull. Rev.* 18 904–909. 10.3758/s13423-011-0131-8 21748418

[B42] McClelland J. L. McNaughton B. L. O’Reilly R. C. (1995). Why there are complementary learning systems in the hippocampus and neocortex: insights from the successes and failures of connectionist models of learning and memory. *Psychol. Rev.* 102 419–457. 10.1037/0033-295X.102.3.419 7624455

[B43] McDaniel M. A. Pressley M. Dunay P. K. (1987). Long-term retention of vocabulary after keyword and context learning. *J. Educ. Psychol.* 79 87–89. 10.1037/0022-0663.79.1.87

[B44] Myréen S. (2017). Evaluating the role of multisensory elements in foreign language acquisition. *Int. J. Educ. Pedagog. Sci.* 11 423–426.

[B45] Oldfield R. C. (1971). The assessment and analysis of handedness: the edinburgh inventory. *Neuropsychologia* 9 97–113. 10.1016/0028-3932(71)90067-4 5146491

[B46] Pishghadam R. (2016). “Emotioncy, extraversion, and anxiety in willingness to communicate in english,” in *Proceedings of the 5th international Conference on language, education, and innovation*, eds Lokman W. A. Fazidah F. M. Salahuddin I. Mohd I. A. W. (London: Infobase Creation Sdn Bhd), 1–5.

[B47] Pishghadam R. Abbasnejad H. (2016). Emotioncy: a potential measure of readability. *Int. Electronic J. Elementary Educ.* 9 109–123.

[B48] Pishghadam R. Shakeebaee G. Shayesteh S. (2018). Introducing cultural weight as a tool of comparative analysis: an emotioncy-based study of social class. *Human. Diliman* 15 1–20.

[B49] Pishghadam R. Shayesteh S. (2016). Emotioncy: a post-linguistic approach toward vocabulary learning and retention. *Sri Lanka J. Soc. Sci.* 39 27–36. 10.4038/sljss.v39i1.7400

[B50] Pishghadam R. Daneshvarfard F. Shayesteh S. (2021a). Oscillatory neuronal dynamics during L2 sentence comprehension: the effects of sensory enrichment and semantic incongruency. *Lang. Cogn. Neurosci.* 10.1080/23273798.2021.1886312

[B51] Pishghadam R. Jajarmi H. Shayesteh S. (2021b). Sense combinations influence the neural mechanism of L2 comprehension in semantically violated sentences: insights from emotioncy. *J. Neurolinguistics* 58:100962. 10.1016/j.jneuroling.2020.100962

[B52] Schneider E. Kulmhofer A. (2016). Helping struggling learners of english as an additional language succeed with interactive multisensory structured strategies. *BELT-Brazilian English Lang. Teach. J.* 7 3–25. 10.15448/2178-3640.2016.1.23215

[B53] Schweinberger S. R. Neumann M. F. (2016). Repetition effects in human ERPs to faces. *Cortex* 80 141–153. 10.1016/j.cortex.2015.11.001 26672902

[B54] Shayesteh S. (2019). *The Neurocognitive Effects of Foreign Language Comprehension in Response to the Emotioncy-based Language Instruction (EBLI): Evidence from Event-related Brain Potentials (ERPs) During Semantic Processing of a Sentence.* Doctoral dissertation, Mashhad: Ferdowsi University of Mashhad.

[B55] Shayesteh S. Pishghadam R. Khodaverdi A. (2020). FN400 and LPC responses to different degrees of sensory involvement: a study of sentence comprehension. *Adv. Cogn. Psychol.* 16 45–58. 10.5709/acp-0283-6 32566053 PMC7293998

[B56] Sobel H. S. Cepeda N. J. Kapler I. V. (2011). Spacing effects in real-world classroom vocabulary learning. *Appl. Cogn. Psychol.* 25 763–767. 10.1002/acp.1747

[B57] Sparks R. L. Artzer M. Patton J. Ganschow L. Miller K. Hordubay D. J. (1998). Benefits of multisensory structured language instruction for at-risk foreign language learners: a comparison study of high school Spanish students. *Annals Dyslexia* 48 239–270. 10.1007/s11881-998-0011-8

[B58] Stein B. E. Burr D. Constantinidis C. Laurienti P. J. Alex Meredith M. Perrault T. J. Jr. (2010). Semantic confusion regarding the development of multisensory integration: a practical solution. *Eur. J. Neurosci.* 31 1713–1720. 10.1111/j.1460-9568.2010.07206.x 20584174 PMC3055172

[B59] Subagdja B. Wang W. Tan A. H. Tan Y. S. Teow L. N. (2012). “Memory formation, consolidation, and forgetting in learning agents,” in *Proceedings of the AAMAS*, (New York, NY: ACM), 1007–1014.

[B60] Talsma D. Senkowski D. Soto-Faraco S. Woldorff M. G. (2010). The multifaceted interplay between attention and multisensory integration. *Trends Cogn. Sci.* 14 400–410. 10.1016/j.tics.2010.06.008 20675182 PMC3306770

[B61] Talsma D. Woldorff M. G. (2005). Selective attention and multisensory integration: multiple phases of effects on the evoked brain activity. *J. Cogn. Neurosci.* 17 1098–1114. 10.1162/0898929054475172 16102239

[B62] Tanner D. Grey S. van Hell J. G. (2017). Dissociating retrieval interference and reanalysis in the P600 during sentence comprehension. *Psychophysiology* 54 248–259. 10.1111/psyp.12788 27859315

[B63] Tanner D. Nicol J. Brehm L. (2014). The time-course of feature interference in agreement comprehension: multiple mechanisms and asymmetrical attraction. *J. Memory Lang.* 76 195–215. 10.1016/j.jml.2014.07.003 25258471 PMC4170797

[B64] Van Berkum J. J. Brown C. M. Zwitserlood P. Kooijman V. Hagoort P. (2005). Anticipating upcoming words in discourse: evidence from ERPs and reading times. *J. Exp. Psychol. Learn. Mem. Cogn*. 31, 443–467. 10.1037/0278-7393.31.3.443 15910130

[B65] Wills K. M. Liu J. Hakun J. Zhu D. C. Hazeltine E. Ravizza S. M. (2017). Neural mechanisms for the benefits of stimulus-driven attention. *Cereb. Cortex* 27 5294–5302. 10.1093/cercor/bhw308 28334189

[B66] Wirebring L. K. Wiklund-Hörnqvist C. Eriksson J. Andersson M. Jonsson B. Nyberg L. (2015). Lesser neural pattern similarity across repeated tests is associated with better long-term memory retention. *J. Neurosci.* 35 9595–9602. 10.1523/JNEUROSCI.3550-14.2015 26134642 PMC6605150

